# A Meeting of the Minds on Mice

**DOI:** 10.1289/ehp.114-a406

**Published:** 2006-07

**Authors:** Ernie Hood

If genetics research is ever to fulfill its promise of revolutionizing medicine, genotypes must be linked to phenotypes—that is, individual genomic characteristics must be identified and associated with outcomes in the forms of disease susceptibility and/or development; individual responses to drugs, infectious agents, or environmental exposures; or other individual characteristics such as behavioral tendencies. Why does the person who never smoked develop lung cancer, while the three-pack-a-day smoker remains healthy? Why do some people become addicted to drugs, while other users are never hooked? Why does a particular medication work wonders in some people, but not work at all in others? These and countless similar questions represent the enormous challenge still facing researchers as they strive to make personalized medicine a clinical reality.

The answers to many of these questions may yet be discovered in the genomes of mice, our diminutive mammalian relatives. That’s certainly the hope and belief of the members of the mouse genetics community, 180 of whom gathered 6–9 May 2006 in Chapel Hill, North Carolina, for the fifth annual meeting of the Complex Trait Consortium (CTC), a loosely woven international organization tightly knit in its dedication to elucidating human characteristics by identifying their genetic counterparts in mice.

The “complexity” of complex traits derives from the fact that they are polygenic—multiple genes interact to cause these conditions, and the genes involved may not interact additively. Ninety-three reports presented at the CTC meeting updated progress in the hunt for the multiple genes and quantitative trait loci (or chromosomal “hot spots”) associated with a wide variety of complex traits such as heart failure, tumor resistance, obesity, drug and alcohol addiction, and schizophrenia. Sponsored by the NIEHS, the UNC–Chapel Hill, and Agilent Technologies, the conference brought together a diverse group of mouse geneticists, molecular biologists, statisticians, and bioinformaticists from 10 countries.

The CTC is all about collaboration and interaction. “It’s unquestionably the best meeting that I go to every year,” says Abraham Palmer, an assistant professor of human genetics at the University of Chicago. “The opportunities to communicate with other geneticists working in other fields and with the people who develop our methodology are critically important, and accelerate by months or even years the rate at which the field can move forward,” he says.

Karlyne Reilly, a principal investigator in the Mouse Cancer Genetics Program at the National Cancer Institute, agrees. “It brings together a wide variety of science around techniques and how you solve the problems that are common to these different areas,” she says. “I always come away with new tools to play with, that I can apply to my own research.”

## Building a Better Mouse Line

The CTC is presently at the midpoint of building a resource that should prove enormously valuable in the effort to associate genotypes with phenotypes. The Collaborative Cross (CC) is a carefully planned and controlled mouse recombinant inbreeding program that began in 2005 with eight genetically heterogeneous strains. Upon its expected completion in about four years, 1,000 lines closely modeling the breadth of human genetic diversity will have been generated. According to conference keynote speaker Jean-Louis Guénet, a professor emeritus of mouse genetics at the Institut Pasteur in Paris, it will be “one of the most important pages in the book of genetics of the future.”

Armed with several powerful new bioinformatic and biostatistical tools being developed specifically to take full advantage of the resource, the CC will enable researchers to hunt far more precisely and efficiently for the multiple genes and quantitative trait loci that constitute complex traits, and will allow the community to share and integrate their raw data sets far more effectively.

“The idea is to accumulate as much diverse data as possible for relatively fixed strains, what we call the ‘genetic reference population,’” says Robert Williams, a professor of anatomy and neurobiology at the University of Tennessee Health Science Center and one of the founders of the CTC. “The hope is that everybody will use their own tools—their own methods and their own phenotypes—but the Collaborative Cross will provide a way to bind those results together by using the same animal resource.”

According to conference co-organizer David Threadgill, an associate professor of genetics at UNC–Chapel Hill, the goal is for the CC to evolve to become “the central resource for experimental mammalian biology.” With a fixed genetic reference population and common tools, he says, “it will be the resource that everybody turns to,” because every piece of data collected through the CC will be immediately comparable to any other piece of data in the database.

## CC Riding

The CC will enable a so-called systems genetics approach, as opposed to the traditional, laborious effort to identify one gene at a time. As Guénet points out, diseases that are the consequences of the alteration of a single gene—one example is cystic fibrosis—tend to be marginal in terms of frequency. However, polygenic diseases tend to be much more widespread, he says: “Next door to you, you probably have someone with asthma, dermatitis, or autoimmune disease. . . . So we have to work hard to understand the genetic determinants of these complex diseases, and presumably what we are going to learn from the mouse can be transposed to the human being, because we share ninety-eight percent of our genes with the mouse.”

Williams shares Guénet’s optimism about the tremendous potential of the CC to shed useful light on common human diseases. “You have to understand the function of the gene and its products in a complex milieu, in a mouse or human—not only a mouse or human, but many different mice and many different humans,” he says. “We think [the CC] will provide the resource to do that.” He adds that the ability to conduct experimental population-based research with the CC should allow much more comprehensive exploration of the genetics associated with gene–environment interactions.

That exploration will also be enhanced by the completion of a mouse genetics initiative undertaken by the NIEHS and Perlegen Sciences to identify the genetic variants in 15 diverse strains of laboratory mice, including SNPs (single-nucleotide polymorphisms), indels (insertions/deletions), and haplotypes (blocks of related SNPs). The database, a project of the recently established NIEHS Center for Rodent Genetics, is scheduled to be unveiled in September 2006, and is anticipated to be a rich and robust source of information for the mouse genetics community.

Signs of early but significant progress in the CC initiative were among the highlights of the meeting. Conference co-organizer John E. French, an NIEHS research physiologist, is encouraged by results emerging from pilot studies. “There’s at least been a proof of principle established that it’s going to be a very effective tool,” he says. “We are only seeing the beginning evidence of that—there’s a long way to go—but some of the promise has been identified and, I think, validated.” According to Williams, the pilot project is now of sufficient size (two recombinant inbred sets, LXS and BXD, with 80 member strains) that “it provides the community with a good flavor of what this will look like when we have an order of magnitude more strains than we do now.”

Threadgill is excited by the flavor that’s already emerging. “The major things that are starting to come out are the results of integrating data sets, integrating genetic variations, and integrating gene expression patterns,” he says. The new knowledge that’s coming out of that—the identity of new genes that are potential master modulators of genetic networks, and how those may actually also be very important for mediating disease processes—speak to the remarkable potential that will be realized when the CC is completed.

## A Case in Point

Research results presented by Palmer on his group’s work at the University of Chicago illustrate the broad outlines of the types of studies being undertaken by mouse geneticists. Palmer and colleagues are investigating the genetic underpinnings of susceptibility or resistance to drug addiction; given today’s working definition of “the environment,” recreational drug use is fast becoming a xenobiotic exposure of great interest. An understanding of the genotypic differences between addiction susceptibility and resistance could lead to new targets for therapeutic drugs or preventive interventions.

The team selectively bred mice to have very high or very low sensitivity to locomotor stimulation, a particular behavioral effect of methamphetamine that is a characteristic animal response to drugs of abuse. They then measured the expression of more than 14,000 genes in a region of the animals’ brain known to be involved in response to the drug. Ultimately, they arrived at a candidate gene that was found to be very differentially expressed in the high- and low-sensitivity mice—casein kinase 1 epsilon (*Csnk1e*). It was a gene already known to be involved in locomotor stimulant response of animals to various drugs. But the question then became, was it important in humans?

Fortuitously, thanks to colleague Harriet de Wit of the University of Chicago Department of Psychiatry, Palmer had access to DNA from a cohort of 100 healthy human volunteers. In a double-blind study, the subjects received 0-, 10-, and 20-mg doses of amphetamine in a randomized order. Responses were measured by standardized questionnaires, and were then compared to results of genotyping tests, to see whether there was a correlation between response to the drug and polymorphisms in *Csnk1e*.

“We found a statistically significant association between this gene, *Csnk1e*, and people’s sensitivity to the euphoric effects of the drug,” says Palmer. “So the people with one genotype ‘got a buzz,’ while people with another genotype didn’t. We hypothesize that that may have implications for the likelihood of a person with one genotype who samples the drug to continue to use the drug, and that of course would put them at grave risk for developing an abusive relationship with the drug.”

Palmer suspects that polymorphisms in *Csnk1e* may also be important in a variety of other systems whose mechanisms might be similar to that of addiction. These include the manic phase of bipolar disorder and the use of stimulants to treat attention deficit/hyperactivity disorder.

“I think we’re now at a point where it’s just about to become easy to go from a phenotype to identifying some of the genes that are involved in that phenotype,” says Palmer. “To get all of them is going to take longer, and it’s going to require further refinements in our methodology, but I actually think that the story I told is going to become a common story. . . . In the same way that molecular biology took a long time to mature, and now is unbelievably central to the way we think about the progress of medicine and health sciences, I think this field of genetics is right at that turning point.”

Knowledge gained from the genomes of our mammalian cousins by groups like the CTC may provide the vital information to eventually usher in the much-anticipated era of personalized medicine. Says Threadgill, “What it really comes down to is being able to predict which individuals are going to be susceptible to certain environmental exposures or disease processes, which individuals are going to respond adversely to combinations of alleles, so that interventions and preventive medicine can be applied where they need to be applied, rather than in global fashion.”

## Figures and Tables

**Figure:**
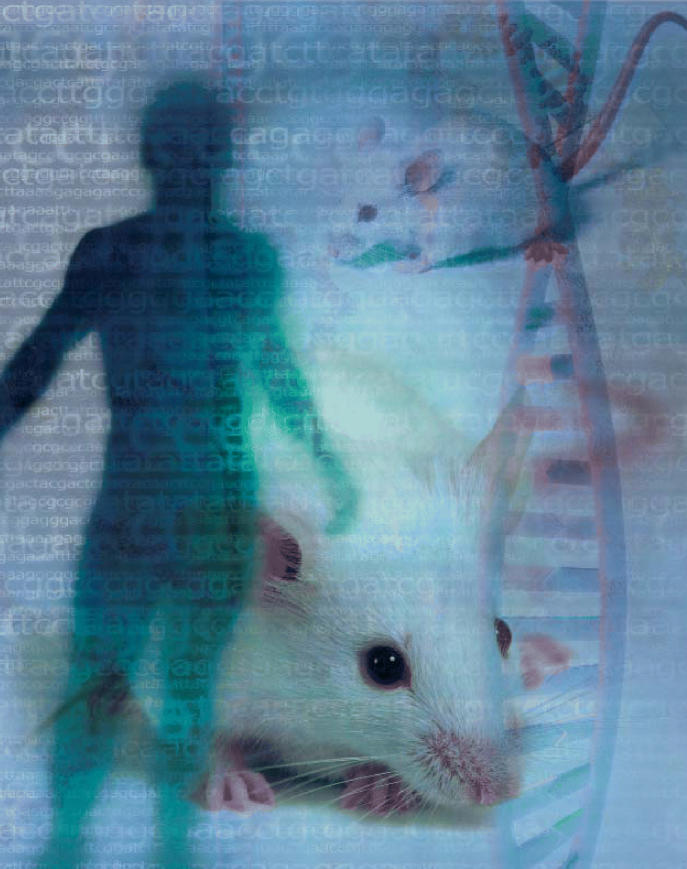
Culling out complex traits A consortium of international scientists is launching new mouse research initiatives to help elucidate genetic components of complex human diseases.

